# Network dynamics with BrainX^3^: a large-scale simulation of the human brain network with real-time interaction

**DOI:** 10.3389/fninf.2015.00002

**Published:** 2015-02-24

**Authors:** Xerxes D. Arsiwalla, Riccardo Zucca, Alberto Betella, Enrique Martinez, David Dalmazzo, Pedro Omedas, Gustavo Deco, Paul F. M. J. Verschure

**Affiliations:** ^1^Synthetic Perceptive Emotive and Cognitive Systems Lab, Center of Autonomous Systems and Neurorobotics, Universitat Pompeu FabraBarcelona, Spain; ^2^Computational Neuroscience Group, Center for Brain and Cognition, Universitat Pompeu FabraBarcelona, Spain; ^3^Institució Catalana de Recerca i Estudis AvançatsBarcelona, Spain

**Keywords:** connectomics, virtual reality, neural dynamics, large-scale brain networks, big data, virtual neurosurgery

## Abstract

BrainX^3^ is a large-scale simulation of human brain activity with real-time interaction, rendered in 3D in a virtual reality environment, which combines computational power with human intuition for the exploration and analysis of complex dynamical networks. We ground this simulation on structural connectivity obtained from diffusion spectrum imaging data and model it on neuronal population dynamics. Users can interact with BrainX^3^ in real-time by perturbing brain regions with transient stimulations to observe reverberating network activity, simulate lesion dynamics or implement network analysis functions from a library of graph theoretic measures. BrainX^3^ can thus be used as a novel immersive platform for exploration and analysis of dynamical activity patterns in brain networks, both at rest or in a task-related state, for discovery of signaling pathways associated to brain function and/or dysfunction and as a tool for virtual neurosurgery. Our results demonstrate these functionalities and shed insight on the dynamics of the resting-state attractor. Specifically, we found that a noisy network seems to favor a low firing attractor state. We also found that the dynamics of a noisy network is less resilient to lesions. Our simulations on TMS perturbations show that even though TMS inhibits most of the network, it also sparsely excites a few regions. This is presumably due to anti-correlations in the dynamics and suggests that even a lesioned network can show sparsely distributed increased activity compared to healthy resting-state, over specific brain areas.

## 1. Introduction

How should one visualize and simulate the large amounts of data being generated nowadays in neurobiology, in ways that could inform our understanding of the structure and function of the brain? Would that also link to clinical applications? Over the years, the cumulative spate of studies in structural and functional neuroimaging, electrophysiology, genetic imaging and axonal-tracing studies have generated enormous amounts of data (found in online repositories such as http://www.neuroscienceblueprint.nih.gov/connectome/ and http://www.brain-map.org to name a few), which, on one hand have led to many insights on the intricate patterns of signaling and connectivity, as well as the existence of multi-scale processes in the brain; on the other hand, it has exposed the need for an integrative framework for modeling and simulating whole-brain dynamics and function. To address this need, neuroscientists started using ideas from network engineering, thinking of the brain as a complex dynamical network of neurons, thus giving rise to the field of brain connectomics (Hagmann, 2005; Sporns et al., 2005). Akin to the genome, which is a map of the genetic sequence of an organism, the connectome is a map of the neuronal circuitry of an organism emphasizing the nodes and their connections where nodes can represent volumes of neuronal tissue to single neurons. Of course, merely a map of this network is not sufficient to predict or understand function. Being a dynamic network, signaling processes in the brain operate across a range of spatial and temporal scales. Therefore, a mechanistic understanding of these processes is essential to gain insight into cognitive function. At the same time, the complexity of the connectome means that these signaling circuits cannot be understood in isolation or even in a serial manner, but necessarily have to be seen in the functional context of the whole network. This calls for a large-scale network level analysis and simulation of whole-brain activity and an associated immersive visualization and interaction system. This is the challenge BrainX^3^ aims to tackle.

BrainX^3^ is a large-scale simulation of the human cerebral connectome, which uses both anatomical structure and biophysical dynamics in order to reconstruct activity and predict function. Structural connectivity of the network is obtained from human Diffusion Spectrum Imaging (DSI) data (Hagmann et al., 2008). Each node of the connectivity matrix corresponds to a population of neurons. For simulating dynamics, BrainX^3^ offers the user a choice of dynamical models that can be implemented, namely, population dynamics based on a linear-threshold transfer function, or a non-linear sigmoidal transfer function with decay, or dynamical mean-field models (Wong and Wang, 2006; Deco et al., 2013). The last of these models are the most interesting as they come closest to biology; they compute aggregate neural activity taking into account synaptic dynamics and stochasticity.

Until the advent of the connectome (Hagmann, 2005; Sporns et al., 2005), the traditional method of choice for investigating non-invasive human resting-state dynamics has heavily relied on back-inference of neural mechanisms from BOLD signals. Despite the many successes of this approach, a few drawbacks remain; for instance, it does not provide precise temporal information on the flow of activity through the network, and being a signal based on haemodynamic responses, it is at best only a proxy for neural activity. However, the birth of the field of connectomics has helped turn this around. Combining structural connectivity data with detailed neuronal dynamics made it possible to instead predict functional activity (Honey et al., 2009). These predictions have been validated for both spiking neuron as well as dynamical mean-field models, when compared to empirical BOLD data (Deco et al., 2013). In this work we implement mean-field dynamics in BrainX^3^ using Hagmann et al.'s DSI network to reconstruct neurodynamic activity for the entire cortex within a 3D virtual environment, for the purpose of investigating temporal patterns of whole-brain neural activity when the brain is in the resting-state. The resting-state refers to the state of spontaneous neural activity recorded in the absence of any specific tasks being instructed to the subject. In computational models, this corresponds to no specific (or localized) external input currents being injected into the network above the overall baseline current. The activity in this spontaneous state is far from random. Hence, understanding the biophysical dynamics of the resting-state constitutes an important challenge.

**Figure 1:**
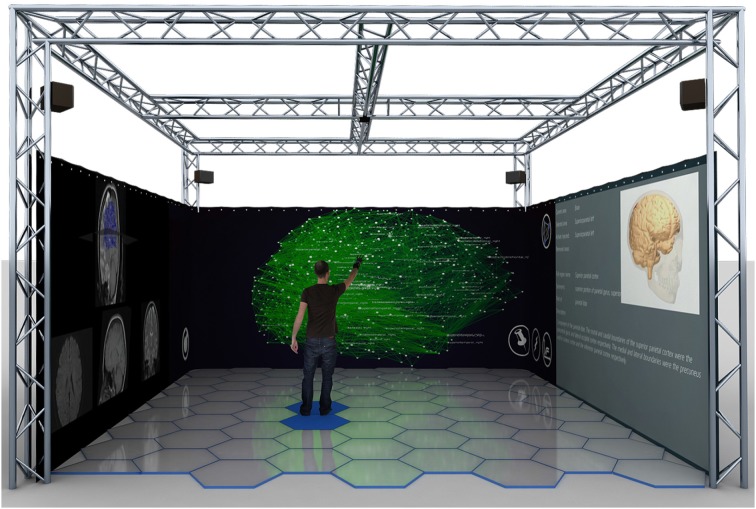
**Computer rendering of BrainX^3^ within the eXperience Induction Machine (XIM)**. The 3D connectome network and its simulated dynamics are projected on the frontal screen. The screen on the right displays regional information of selected brain areas from a curated database, while the left screen shows 2D axial slices of the brain and indicates regions of activity. The user can navigate and interact with the model with predefined hand gestures.

The technology of BrainX^3^ is layered on four modular components: an input module, a data processing module, a visualization and interaction module, and a simulation and analysis module. The integration of these modules generates the full user experience. Simulations run in the eXperience Induction Machine (XIM), an enclosed virtual/mixed reality chamber, which enables user-immersion and exploration of virtually rendered scenarios (refer to Figure 1) (Bernardet et al., 2010; Betella et al., 2014c). Based on DSI data, a 3D model of the connectome network is reconstructed within the XIM, providing users with an inside-out perspective of the brain connectome and allowing them to navigate through pathways in the network. Additionally, the XIM supports a custom-built large-scale neural simulator, iqr, which communicates bi-directionally with the virtual brain network and imposes dynamics on it (Bernardet and Verschure, 2010). Furthermore, BrainX^3^ is based on a natural user interaction paradigm, such that using natural gestures, i.e., hand gestures, body posture, etc., users can navigate the virtual space, select and bookmark brain areas, perform surgeries, stimulate any region of the network to investigate the dynamics of the neuronal activity reverberating through associated areas. Finally, for data analysis, BrainX^3^ communicates bi-directionally, using standard protocols such as UDP or YARP (Metta et al., 2006), with external network analysis tools, including the Brain Connectivity Toolbox (BCT) (Rubinov and Sporns, 2010) running on a MATLAB client.

What is BrainX^3^ capable of? It provides the possibility of analyzing and interacting in real-time with the simulated activity. Compared to functional correlations, dynamical analysis of causal activity serves as a powerful tool to unravel mechanisms of large-scale neural circuits. Indeed, coupling structural connectivity data with detailed enough population dynamics should be sufficient in predicting functional correlations and large-scale activity patterns. As examples, we use BrainX^3^ to demonstrate the dynamics of the brain in the resting-state as well as under perturbations due to evoked stimuli. We investigate how neural activity reorganizes following simulated lesions. Additionally, using graph theoretic measures from the BCT we can also determine shortest paths between nodes.

At this point, it is also worth drawing attention to the growing eco-system of ‘big brain projects’ and other neuroinformatics tools which complement BrainX^3^. Among these are the Connectome Workbench of the Human Connectome Project (http://www.humanconnectome.org/connectome/connectome-workbench.html) (Marcus et al., 2013), the Brain Explorer 2 of the Allen Institute for Brain Science (http://www.brain-map.org), the Glass Brain Project (http://neuroscapelab.com/projects/glass-brain/) (Mullen et al., 2013), the VisNEST tool (Nowke et al., 2013) and The Virtual Brain (Jirsa et al., 2010; Sanz-Leon et al., 2013). While many of them are 3D visualization tools, some of them also include dynamics and interaction. In that sense, The Virtual Brain comes closest to the objectives of BrainX^3^, but for example, it does not include real-time interaction. Unlike the aforementioned, BrainX^3^ runs in a completely immersive virtual reality chamber, facilitating real-time interaction with the simulation using natural gestures. For the benefit of the neuroinformatics community, a portable laptop version, including interaction (but without user immersion), is currently under development. Besides, visualization, interaction and simulation, BrainX^3^ has been developed with a vision toward a “smart exploration space for big data” as part of the European Union CEEDS (Collective Experience of Empathic Data Systems) project (http://ceeds-project.eu and http://www.brainx3.com).

## 2. Materials and methods

### 2.1. Hardware and system architecture

The virtual reality environment supporting BrainX^3^ is the eXperience Induction Machine (XIM) (Bernardet et al., 2010; Betella et al., 2014c; Omedas et al., 2014). The XIM is a 25 m^2^ human accessible space (schema in Figure 1) equipped with 360° surround screens, an interactive luminous floor with pressure sensors, a marker-free tracking system, a Kinect^TM^, microphones, a sonification system and wearable sensors, that support human-machine interaction in the exploration of complex datasets. The computational platform running BrainX^3^ in the XIM includes four latest generation machines that communicate bi-directionally using the YARP protocol within a high speed LAN connection: 2 PCs dedicated to graphical rendering (INTEL CORE i7 2600K 3,4GHZ/8mb/LGA1155, two DDR3s 4GB 1333Mhz KINGSTON, AMD FirePro V7900 Professional with AMD Eyefinity technology) with a total of eight display port outputs, each of which is connected to an HD projector (Epson PowerLite Pro G5450WUNL), thus creating a 360° projection display that surrounds the user; 1 server dedicated to sensors recording and real-time interaction (HP proliant DL160 G6, Xeon E5506, 2.13 GHz) that is connected to the XIM sensors and effectors, including a Microsoft Kinect2™, the sonification system and the interactive floor; 1 server dedicated to simulation and computation (HP proliant DL160 G6, Xeon E5506 at 2.13 GHz) that runs the neural network simulator iqr (http://iqr.sourceforge.net) and MATLAB.

**Figure 2:**
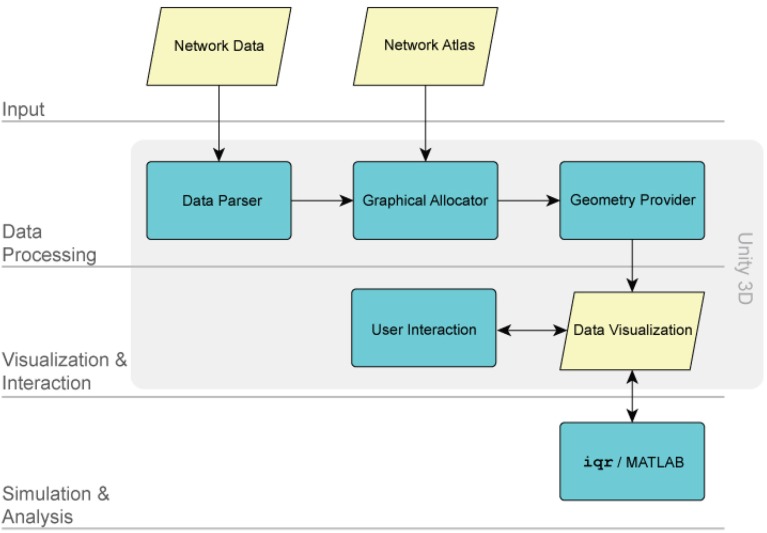
**The BrainX^3^ architecture**. The architecture of BrainX^3^ is designed on four layers: the input module, the data processing module, the visualization/interaction module and the simulation/analysis module. Each layer is further characterized by dedicated sub-modules (represented by the colored boxes). The arrows indicate interaction between different modules and sub-modules.

Within the XIM virtual reality environment, BrainX^3^ functions as a data visualization and simulation tool. The processing architecture of BrainX^3^ is schematically illustrated in Figure 2. The input module (layer) has two components: the *network data* and the *network atlas*. The *network data* is the connectome dataset, while the *network atlas* contains the coordinates of each element of the network. Both of these are stored in GraphML (XML) format. The *data parser* generates a data structure and specifies the components of the graph. The *graphical allocator* reads the meta data associated to each one of the elements that compose the network and associates them to the 3D coordinate system, included in the *network atlas*. In BrainX^3^, we adopt the standard anatomical coordinates of the Talairach atlas (Talairach and Tournoux, 1988), however other coordinate systems can just as easily be applied. The *geometry provider* plots the results as a 3D representation of the data by combining the instances generated by the parser with the coordinates specified within the atlas. The components responsible for data processing follow a *Model-View-Controller* (MVC) design pattern. Both data processing and real-time rendering have been developed and implemented using Unity 3D (http://unity3d.com/). The advantage of such a modular structure is that it provides BrainX^3^ with the adaptability for visualization and simulation of other data types besides neural data, which can be stored as a network or organized in a hierarchical structure, such as gene regulatory networks, social networks, etc.

### 2.2. Visualization and simulation

Visualization and reconstruction of the connectome within BrainX^3^ is based on DSI data of white matter fiber structural connectivity averaged from five healthy right-handed male human subjects (Hagmann et al., 2008). The dataset contains 998 voxels (nodes) belonging to 33 cortical areas per hemisphere (refer Table 1), for a total of 66 areas. The 998 Regions of Interest (ROIs) have an average size of 1.5 cm^2^ and each ROI is associated with {*x*, *y*, *z*} coordinates as per the Talairach coordinates of ROIs (Talairach and Tournoux, 1988). Since tractography does not determine the directionality of the fibers, the connectivity matrix (approximately 17000 bi-directional connections) is symmetric at the ROI level. Connection strengths within the network refer to normalized number of white matter fiber tracts between ROIs.

**Table 1:** **33 brain regions on each hemisphere (ID), abbreviated name (Abbr.), anatomical name (Brain region) and ROI node numbers for each region on right (R) and (L) hemispheres**.

**ID**	**Abbr**.	**Brain region**	**Nodes (R)**	**Nodes (L)**
1	BSTS	Bank of the superior temporal sulcus	463–469	961–965
2	CAC	Caudal anterior cingulate cortex	191–194	692–695
3	CMF	Caudal middle frontal cortex	126–138	628–640
4	CUN	Cuneus	335–344	835–842
5	ENT	Entorhinal cortex	419–420	918–920
6	FP	Frontal pole	26–27	527–528
7	FUS	Fusiform gyrus	391–412	890–911
8	IP	Inferior parietal cortex	284–311	787–811
9	IT	Inferior temporal cortex	424–442	925–941
10	ISTC	Isthmus of the cingulate cortex	202–209	703–710
11	LOCC	Lateral occipital cortex	355–373	852–873
12	LOF	Lateral orbitofrontal cortex	1–19	501–520
13	LING	Lingual gyrus	374–390	874–889
14	MOF	Medial orbitofrontal cortex	28–39	529–540
15	MT	Middle temporal cortex	443–462	942–960
16	PARC	Paracentral lobule	175–186	677–687
17	PARH	Parahippocampal cortex	413–418	912–917
18	POPE	Pars opercularis	48–57	548–558
19	PORB	Pars orbitalis	20–25	521–526
20	PTRI	Pars triangularis	40–47	541–547
21	PCAL	Pericalcarine cortex	345–354	843–851
22	PSTC	Postcentral gyrus	210–240	711–740
23	PC	Posterior cingulate cortex	195–201	696–702
24	PREC	Precentral gyrus	139–174	641–676
25	PCUN	Precuneus	312–334	812–834
26	RAC	Rostral anterior cingulate cortex	187–190	688–691
27	RMF	Rostral middle frontal cortex	58–79	559–577
28	SF	Superior frontal cortex	80–125	578–627
29	SP	Superior parietal cortex	257–283	760–786
30	ST	Superior temporal cortex	470–497	966–994
31	SMAR	Supramarginal gyrus	241–256	741–759
32	TP	Temporal pole	421–423	921–924
33	TT	Transverse temporal cortex	498–500	995–998

To introduce dynamics into the visualization, the large-scale multi-level neural networks simulator, iqr (http://iqr.sourceforge.net/), is bi-directionally interfaced to Unity. iqr allows the user to design complex neuronal models through a graphical interface and to visualize, analyze and modify the model's parameters in real-time (Bernardet and Verschure, 2010). The architecture of iqr is modular, providing the possibility to define custom neurons and synapses. iqr can simulate large neuronal systems up to 500k neurons and connections and can be directly interfaced to external sensors and effectors. In order to enable real-time user interaction with the reconstructed data, user input from Unity is sent to iqr (Arsiwalla et al., 2013). The neuronal simulator computes the processes and broadcasts the output of the simulation back to the Unity engine in the XIM. The simulation runs with iqr receiving commands through Unity at any time during the simulation. Upon receiving input from iqr, Unity updates the visualized population activity on each node. In its current form, BrainX^3^ can accommodate networks of up to 4000 nodes (albeit with a slower simulation time).

### 2.3. Dynamical models in BrainX^3^

As the connectivity data currently being used by BrainX^3^ is derived from neuroimaging sources, it is more appropriate to model network dynamics by means of neuronal population models. At present, BrainX^3^ allows to run simulations with either of the three models: (i) the linear-threshold model, (ii) non-linear (sigmoidal) model and (iii) dynamical mean-field model. The linear-threshold model simply sums up all the input signals to a population module from various dendrites (within a fixed time window) and fires an output signal to neighboring modules only if the summed inputs cross a designated threshold. Additionally each neuronal population module is stochastic, having Gaussian noise. This was demonstrated in earlier work (Arsiwalla et al., 2013). The non-linear model is similar to above except that the linear-threshold filter is replaced by a sigmoidal filter with decay. This was used in Betella et al. (2014b).

The dynamical mean-field model is a mathematical reduction of a spiking attractor network consisting of integrate and fire neurons with excitatory and inhibitory synapses (Wong and Wang, 2006; Deco et al., 2013). Global brain dynamics of the network of interconnected local networks is described by the following set of differential equations derived in Deco et al. (2013):

(1) $$\frac{\text{d}S_{i}}{\text{d}t}=-\frac{S_{i}}{\tau_{s}}+\left(1-S_{i}\right)\gamma H\left(x_{i}\right)+\sigma v_{i}\left(t\right)$$

(2) $$H(x_{i})=\frac{ax_{i}-b}{1-\text{exp}\left(-d\left(ax_{i}-b\right)\right)}$$

(3) $$x_{i}=wJ_{N}S_{i}-GJ_{N}\sum_{j}C_{ij}S_{j}+I_{0}$$

where *H*(*x_i_*) and *S_i_* respectively correspond to the population rate and the average synaptic gating variable at each local node *i*, *w* is the local recurrent excitation, *G* is a global scaling parameter, *C_ij_* is the matrix of structural connectivity expressing the neuroanatomical connections between areas *i* and *j*, *v_i_* is uncorrelated Gaussian noise. All parameters values, with the exception of σ, which was systematically varied in the present simulation study, are as in Deco et al. (2013) and have been summarized in Table 2.

**Table 2:** **List of parameters of the dynamical mean-field model implemented in BrainX^3^ from Deco et al. (2013)**.

**Parameter**	**Description**	**Value**	**Unit measure**
*N*	Number of nodes	998	
*w*	Local recurrent excitation	0.9	
*a*	Input-output function	270	n/C
*b*	Input-output function	108	Hz
*d*	Input-output function	0.154	s
γ	Kinetic parameter	0.641	s
τ*_S_*	Kinetic parameter	100	ms
*J_N_*	Synaptic coupling	0.2609	nA
*I*_0_	Overall external input	0.3	nA
σ	Noise amplitude	0.01, 0.05, 0.07, 0.1	

Among the three types of models described above, mean-field models are the most interesting as they come closest to biology. They compute aggregate neural activity taking into account synaptic dynamics and stochasticity. Hence, in this paper, our simulations will be based on the dynamical mean-field model. However, compared to Deco et al. (2013), where the dynamics was parametrized on 66 regions, in this work we scale the dynamics to 998 ROIs.

### 2.4. Real-time interaction framework

**Figure 3:**
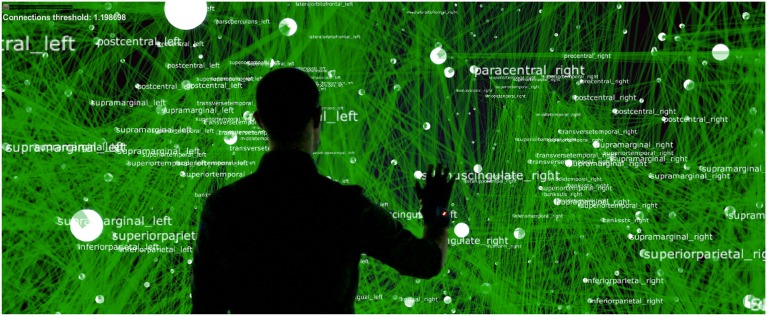
**Interaction within BrainX^3^**. User immersion and interaction within BrainX^3^.

BrainX^3^ allows users to interact in real-time with the simulation (the simulation itself is not real-time, each millisecond of simulation takes between 20 and 50 ms, being slower during interaction). This is a form of on-line interaction, as opposed to a pre-programed off-line mode of interaction. It provides users the possibility to perturb the simulated activity (by injecting currents using predefined hand gestures) mid-way through the run. Gesture recognition and signaling within the XIM is supported via the Social Signal Interpretation (SSI) framework (Wagner et al., 2013) and is based on the Microsoft Kinect^TM^ v2 technology. The Kinect^TM^ detects body joints and two main hand actions: the closed hand and pointing with a finger. All high level mapping and interpretation of gestures is performed by SSI. In order to rotate the network sideways, the user simply has to clench her/his fist and make a sideways arm movement. For zooming into the network, the user moves directly toward the screen and the visualization becomes immersive placing the user “inside” the 3D reconstruction (refer to Figure 3). For stimulating or inhibiting brain areas, the user simply has to control the cursor with a hand movement, select a node or region in the network with a grabbing gesture, then drag and drop the cursor on the icon in the graphical user interface, associated to stimulation or inhibition. This respectively corresponds to injecting external excitatory or inhibitory currents into the dynamics. The strength of the stimulation current is pre-defined in the iqr configuration file (but can be arbitrarily chosen). Stimulations can be performed on one or more brain areas simultaneously. BrainX^3^ then reconstructs reverberating neural activity propagating through the connectome. Furthermore, in order to equip the user with tools for analysis of the outcome of the simulation, BrainX^3^ is also interfaced with the MATLAB Brain Connectivity Toolbox, which enables several graph-theoretic operations to be performed on the reconstructed network such as finding the shortest path between any two nodes or detecting community structure in the data (Rubinov and Sporns, 2010). BrainX^3^ also includes customized interaction functionalities that allow the user to bookmark areas of interest, to tag and visually highlight chosen pathways, to filter network complexity and to model lesions by disabling nodes in order to obtain altered activity associated to the lesion.

## 3. Results

We now put to test the functional capabilities of BrainX^3^ to gain valuable insights on the large-scale dynamics of the human connectome. We start by simulating the global dynamics of the resting-state. Then we lesion the structural network and study aberrant cortical activity for both focal lesions as in stroke patients as well as for diffuse lesions as in multiple sclerosis patients. Next, we study the effect of external perturbations such as trans-cranial magnetic stimulations on the network and its resulting evoked activity. Finally, we demonstrate an exercise in tracing pathways within the cortex in order to extract functional circuits as well as to analyze them. Videos explicitly demonstrating these results in BrainX^3^ have been uploaded on the following link: https://www.youtube.com/playlist?list=PL-BcYpSz98wqVAKuI-ymqDII-6nXK_8uq.

### 3.1. Dynamics of the resting-state

**Figure 4:**
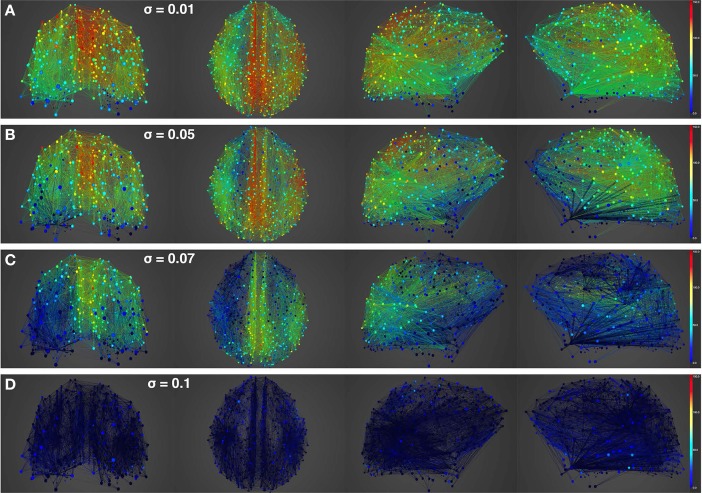
**Simulation of resting-state activity vs. noise showing that increased noise suppresses network activity**. Snapshot of resting-state neural activity at a single time-point (after the dynamics stabilizes around the attractor) for different levels of noise amplitude within BrainX^3^. From top to bottom, noise amplitudes: **(A)** 0.01, **(B)** 0.05, **(C)** 0.07, and **(D)** 0.1. Each row shows screenshots from the posterior, superior and lateral perspectives. The color bar on the right represents neuronal activity in Hz (warmer colors represent higher activation of the nodes). The full simulation can be seen on videos 01 and 02 of the following link: https://www.youtube.com/playlist?list=PL-BcYpSz98wqVAKuI-ymqDII-6nXK_8uq.

**Figure 5:**
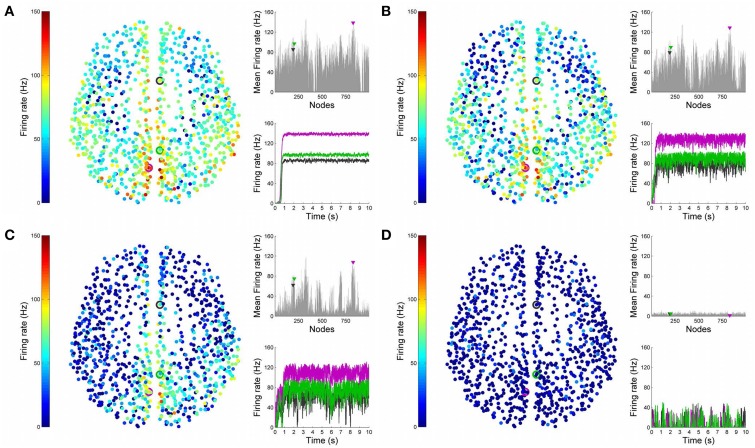
**Analysis of resting-state activity vs. noise**. 2D plots of resting-state neural activity for the same four simulation runs shown in Figure 4. Subplots **(A–D)** respectively refer to noise amplitudes 0.01, 0.05, 0.07, and 0.1. Each subplot shows three graphics: 2D distribution of nodes with activity indicated by colors from the color bar (warmer colors refer to higher activation), mean firing rate for all 998 nodes over the last 2 s of simulation and time-series signals extracted for three seed ROIs rCAC (node 193, shown in black), rISTC (node 205, shown in green) and lPCUN (node 830, shown in magenta).

Figures 4, 5 show results from 10 s of simulation of resting-state dynamics. An important observation made in Deco et al. (2013) was that the resting-state network operates at the edge of a bifurcation. This fixes the global coupling parameter of the model. Analogously, for the scaled model we implement here, the value of the global coupling *G* is determined to be 2.3 using the same observation. Besides that, all other mean-field model parameters (except the noise amplitude σ) are held at exactly the same values as in Deco et al. (2013). The numerics run in time steps of 0.1 ms but we sample data every 1 ms giving 10K points for a run of 10 s. Figure 4 shows screenshots from the front display of BrainX^3^ at the end of four runs of the simulation. Each run was chosen with a different value of noise amplitude, shown in rows A, B, C and D with σ 0.01, 0.05, 0.07, and 0.1 respectively. The four snapshots in each row (from left to right) correspond to the posterior, superior and lateral views respectively. Since BrainX^3^ is interfaced to MATLAB via YARP/UDP, in addition to the 3D reconstruction, we also obtained time-series data that can be analyzed using any statistical tool. This is shown in Figure 5. This analysis was performed off-line using MATLAB. Each of the four subplots A, B, C, and D refer to the same four noise levels. Further, each subplot includes three graphics: a 2D distribution of ROIs with colored nodes indicating activity level at the end of the simulation, a plot showing the mean firing rate of every ROI over the last 2 s and a plot showing the full time-series signal of three randomly chosen nodes. The mean firing activity represents the stable fixed point of the dynamics and in fact the attractor of the resting-state network. The seed ROIs corresponding to the three time-series signals refer to nodes 193, 205, and 830 located in regions rCAC (black), rISTC (green) and lPCUN (magenta) respectively. Table 1 shows the mapping of ROI identities to anatomical region names.

An interesting insight that we gain upon comparing the results of these simulations is the way noise affects the network attractors themselves. This is summarized in the histogram on the left-hand side of **Figure 11**, showing the total mean firing rate of the resting-state network integrated over all ROIs. Each column of the histogram refers to a given noise amplitude. What is interesting is that rather than jumping into a hyperactive or chaotic state, upon increasing intrinsic noise, the dynamics of the network seems to quiet down. For σ 0.07, mean activity for each node is around 40 Hz and for σ 0.1, it almost goes to zero. Remarkably, this happens without the use of any ROI to ROI inhibitory connections. Noise seems to reverse the stability of the previously unstable low firing attractor state.

### 3.2. Dynamics of stroke and multiple sclerosis

**Figure 6:**
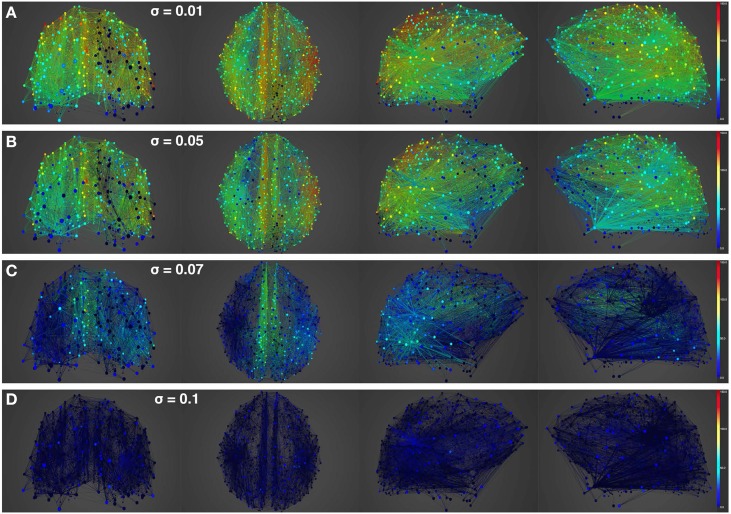
**Simulation of lesioned (focal) brain activity vs. noise**. Snapshot of neural activity at a single time-point in BrainX^3^ following a focal lesion in areas rCUN, rLOCC, and rPCUN. From top to bottom, noise amplitudes: **(A)** 0.01, **(B)** 0.05, **(C)** 0.07, and **(D)** 0.1. Each row shows screenshots from the posterior, superior and lateral perspectives. The color bar on the right represents neuronal activity in Hz (warmer colors represent higher activation of the nodes and lesioned nodes are shown in black). The full simulation can be seen on videos 03 and 04 of the following link: https://www.youtube.com/playlist?list=PL-BcYpSz98wqVAKuI-ymqDII-6nXK_8uq.

**Figure 7:**
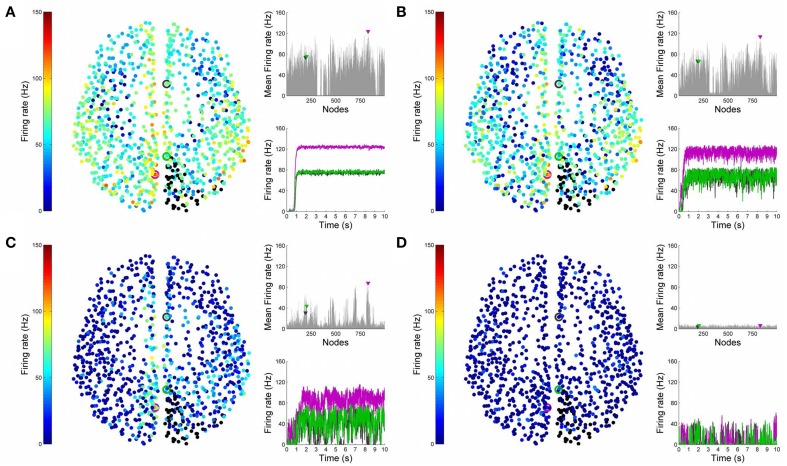
**Analysis of lesioned (focal) brain activity vs. noise**. 2D plots for neural activity following a focal lesion in areas rCUN, rLOCC and rPCUN for the same four simulation runs shown in Figure 6. Subplots **(A–D)** respectively refer to noise amplitudes 0.01, 0.05, 0.07, and 0.1. Each subplot shows three graphics: 2D distribution of nodes with activity indicated by colors from the color bar (warmer colors refer to higher activation and lesioned nodes are shown in black), mean firing rate for all nodes over the last 2 s of simulation and time-series signals extracted for the three seed ROIs rCAC (node 193, shown in black), rISTC (node 205, shown in green) and lPCUN (node 830, shown in magenta).

**Figure 8:**
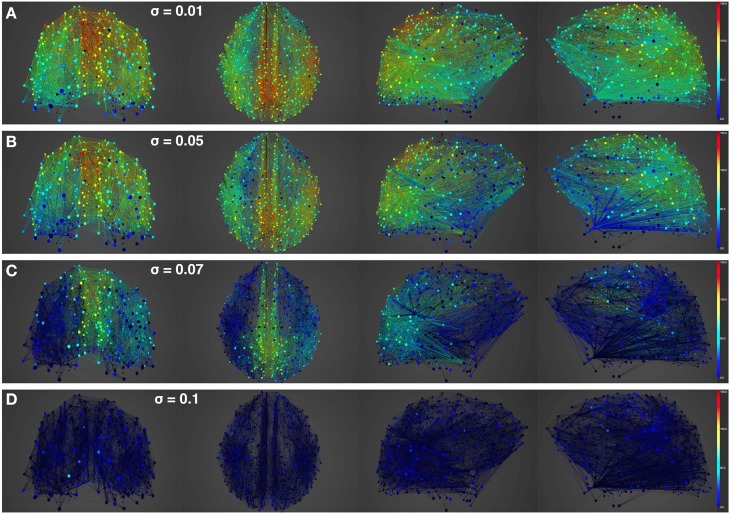
**Simulation of lesioned (diffuse) brain activity vs. noise**. Snapshot of neural activity at a single time-point in BrainX^3^ following a diffuse lesion. The lesion was simulated by disconnecting 50 randomly selected nodes. From top to bottom, noise amplitudes: **(A)** 0.01, **(B)** 0.05, **(C)** 0.07, and **(D)** 0.1. Each row shows screenshots from the posterior, superior and lateral perspectives. The color bar on the right represents neuronal activity in Hz (warmer colors represent higher activation of the nodes and lesioned nodes are shown in black).

**Figure 9:**
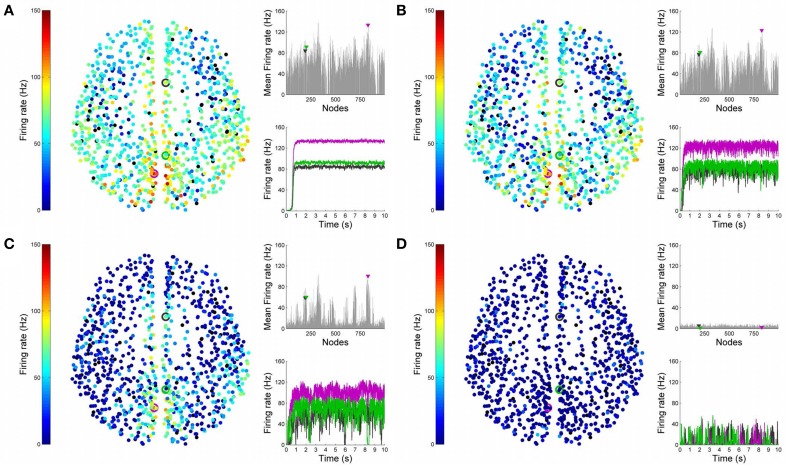
**Analysis of lesioned (diffuse) brain activity vs. noise**. 2D plots for neural activity following a diffuse lesion for the same four simulation runs shown in Figure 8. Again, subplots **(A–D)** respectively refer to noise amplitudes 0.01, 0.05, 0.07, and 0.1. Each subplot shows three graphics: 2D distribution of nodes with activity indicated by colors from the color bar (warmer colors refer to higher activation and lesioned nodes are shown in black), mean firing rate for all nodes over the last 2 s of simulation and time-series signals extracted for the three seed ROIs rCAC (node 193, shown in black), rISTC (node 205, shown in green) and lPCUN (node 830, shown in magenta).

Having looked at the healthy resting-state network above, we now show how lesions can be simulated in BrainX^3^. We consider two lesion types, (i) focal lesions, which occur in the case of stroke patients, and (ii) diffuse lesions, which typically occur in patients with multiple sclerosis. Figures 6, 7 shows results for the former lesion type with the same four levels of noise as above. Figures 8, 9 shows results with diffuse lesions. The focal lesion is constructed on the right hemisphere by severing all white matter fibers connections from all nodes in regions rCUN, rLOCC, and rPCUN. These are a total of 52 disconnected ROIs, amounting to 6.64% of the total connections. The diffuse lesions are constructed by randomly disconnecting individual ROIs distributed throughout the network. To compare with the focal case, we chose 50 scattered ROIs, which amount to 4.91% of the total connections.

**Figure 10:**
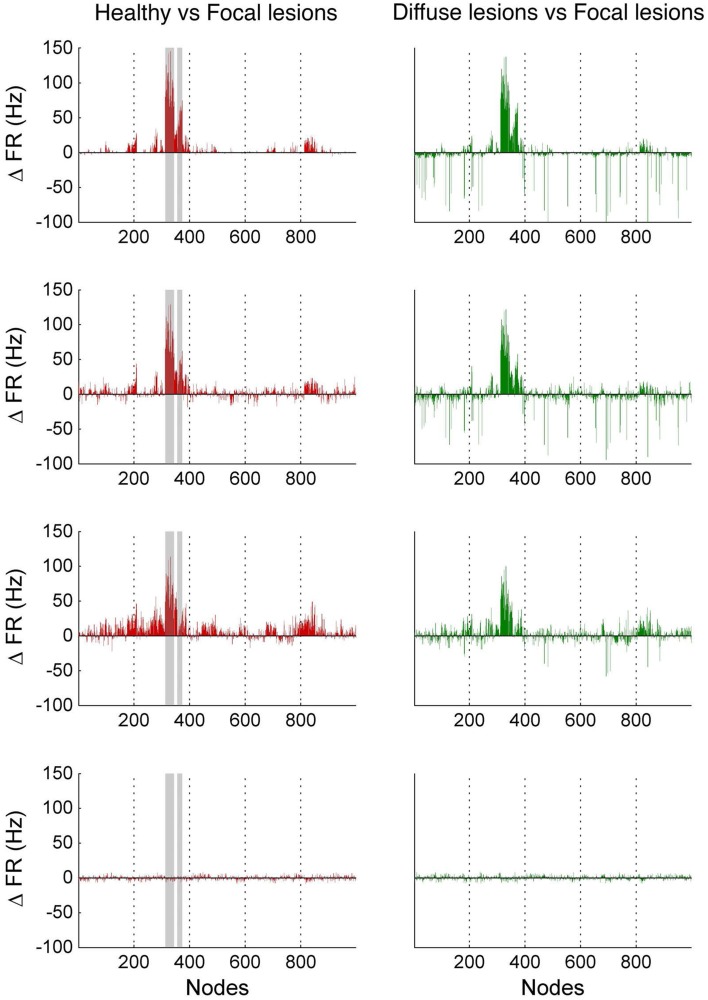
**Firing rate differences Δ FR between healthy and lesioned brains**. The left-hand side shows the difference in mean firing rate between the healthy and focally lesioned networks (plotted on the y-axis) in the attractor state at each ROI (plotted on the x-axis), for the four noise amplitudes (in increasing order, starting at 0.01 on top to 0.1 at the bottom). The gray columns in the figures indicate the lesioned areas rCUN, rLOCC, and rPCUN. The right-hand side shows the same difference but for the diffuse vs. the focally lesioned network.

**Figure 11:**
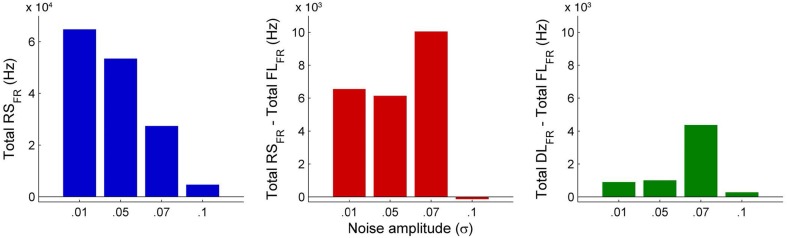
**Comparison of total mean firing rates between healthy and lesioned brains**. The histogram on the left shows the total mean firing rate of the healthy resting-state network (Total RS_*FR*_), integrated over all ROIs, for each value of noise amplitude. The histogram at the center shows the total difference in mean firing between the healthy and focally lesioned networks (Total RS_*FR*_ - Total FL_*FR*_) for each noise amplitude, integrated over all ROIs and the histogram on the right shows the same difference but for the diffuse vs. the focally lesioned networks (Total DL_*FR*_ - Total FL_*FR*_).

In Figure 10, we compare differences between healthy resting-state activity (Figure 4) and the lesioned activity (Figure 6). The four plots on the left side of Figure 10 show the difference in mean firing rate between the healthy and focally lesioned networks (on the y-axis) in the attractor state at every ROI (on the x-axis), for the four noise amplitudes (in increasing order from top to bottom). From this we see that for the lowest noise amplitude (0.01), the lesion mostly affects activity in its anatomical vicinity. However, by the time we reach noise amplitude 0.07, the span of the network affected by the lesion has dramatically increased. Furthermore, the histogram shown in the center of Figure 11 integrates the differences in mean firing over all ROIs to give the total difference in mean firing between the healthy and focally lesioned network for each noise amplitude. The columns of the histogram are on the positive side of the x-axis (except for noise amplitude 0.1, when the activity in both networks is just noise). By the time the noise amplitude rises to 0.07, the lesioned network dramatically differs from the healthy network in total firing. These observations suggest that noisy networks are less resilient to focal lesions.

On the other hand, a comparison of mean activity between the focally lesioned (Figure 6) and diffuse lesioned (Figure 8) networks is shown in the four plots on the right side of Figure 10. The y-axis denotes the difference in mean firing rate between the diffuse and focally lesioned networks in the attractor state. The x-axis runs over all 998 ROIs. Though the number of disabled nodes in both cases is almost the same, we find activity levels in case of diffuse lesions to be markedly higher than in the case of a focal lesion (of course, both conditions have diminished activity compared to the healthy network). The same thing can be seen from the histogram on the right-hand side of Figure 11, showing the total difference in mean firing between the diffuse and focal lesion activity for each noise amplitude, integrated over all ROIs. Again, the columns are on the positive side of the x-axis. Thus, the connectome network shows more resilience to diffuse rather than focal lesions with the same number of nodes. This is presumably due to the wiring architecture of the brain that allows for alternate passages in order to protect against random abrasions.

### 3.3. Causal efferents of TMS perturbations

**Figure 12:**
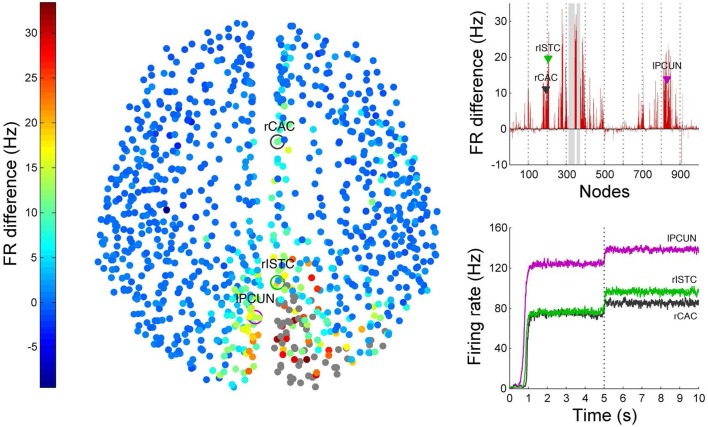
**Results from TMS perturbations applied upon regions rCUN, rLOCC, and rPCUN**. The left figure shows a 2D distribution of nodes with colors indicating difference in mean firing after and before inhibition. The gray nodes indicate regions where TMS is applied. The top right plot quantifies these differences (here the perturbed areas are indicated by the gray columns), while the bottom right plot extracts the firing rate over time for seed nodes 193 (rCAC), 205 (rISTC) and 830 (lPCUN). ROIs in regions rPARC, rCAC, rISTC, rPC, rSP, rIP, and rLING were found to be strongly inhibited as a result of the TMS perturbation in rCUN, rLOCC, and rPCUN. The full simulation can be seen on video 05 of the following link: https://www.youtube.com/playlist?list=PL-BcYpSz98wqVAKuI-ymqDII-6nXK_8uq.

Non-invasive physiological perturbations of specific brain areas using transcranial magnetic stimulations (TMS) have successfully been used for probing neural circuits and their functions. They can be operated either to excite or completely inhibit a given brain area both in the presence or absence of a task. What we want to computationally reconstruct in BrainX^3^ are the causal efferents of the evoked activity due to this stimulation. In Figure 12 we show results for an inhibitory stimulation applied to all the nodes in areas rCUN, rLOCC and rPCUN (the same regions on which we earlier simulated a focal lesion and with network noise amplitude of 0.01). TMS is applied during the first 5 s of the simulation and the network returns to resting-state once stimulation is discontinued. The bottom right plot in Figure 12 shows how this affects the time-series of the same three seed nodes we used (rCAC (black), rISTC (green) and lPCUN (magenta)), which are connected to but not part of the perturbed regions. The change in the firing rate is of the order of 10–20 Hz and upon removing the stimulation, we find that the network returns to resting-state activity in about 40 ± 10 ms. The left diagram in Figure 12 shows all 998 nodes, with the stimulated nodes in gray and the colors in all other nodes denoting the difference in the average firing rate (averaged over 2 s) for each node after and before the perturbation. The averaging is done to take in account variations due to noise. The plot on the top right of Figure 12 shows the exact differences (in red) in average firing rate, after minus before, for each of the 998 nodes (the stimulated areas are shaded in gray), with the black, green and magenta markers referring to the seed nodes. Hence, above the zero difference, we see efferent areas of the network that are inhibited during TMS, whereas, below the zero line refers to efferents that are actually excited during TMS.

**Figure 13:**
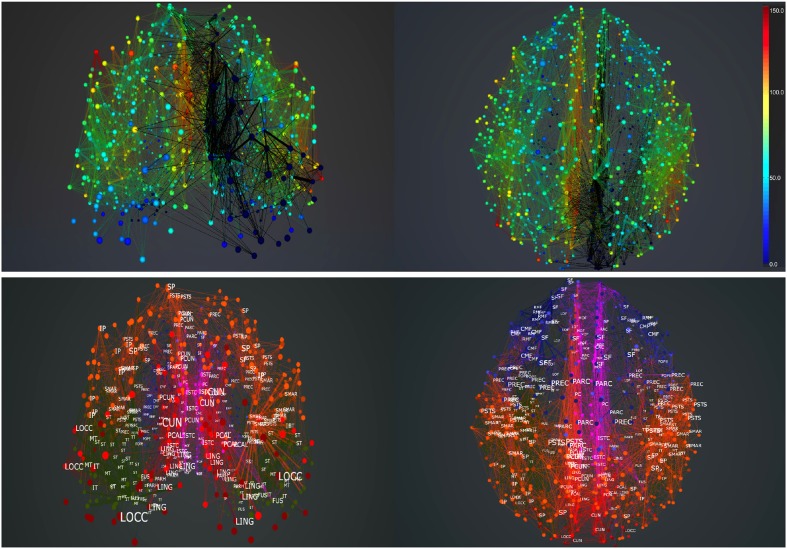
**Pathfinding in the brain**. Extracting efferents of selected regions in BrainX^3^, shown in the top figure. The selected regions are the rCUN, rLOCC, and rPCUN. All paths emerging from these regions are traced in thick black. Screenshots refer to the posterior (top left) and superior view (top right). The bottom figure shows a reference atlas with labels of brain regions in the posterior (bottom left) and superior (bottom right) sides of the network. The colors in the atlas refer to major lobes: frontal (blue), temporal (green), occipital (red), parietal (orange) and cingulate cortex (purple).

Clearly, the results show that areas anatomically closer to the perturbed regions are most affected, but they also show specific long range connections in the frontal, temporal and limbic lobes that are affected by stimulating areas rCUN, rLOCC, and rPCUN (in the occipital and parietal lobes). Figure 12 shows ROIs in regions rPARC, rCAC, rISTC, rPC, rSP, rIP, and rLING are strongly inhibited. Since the stimulated areas here are exactly the same that we lesioned for simulating stroke dynamics, the map of efferents we find after TMS are also part of the affected pathways following the lesion. As described in the next subsection and Figure 13, in BrainX^3^ we can extract these efferents explicitly in a 3D reconstruction.

Though most of the TMS efferents are inhibited during the stimulation phase, interestingly, a small number of them are also excited, showing an average firing rate higher than the non-perturbed (resting-state) value. These occur sparsely in regions rLOF, rRMF, rCMF, lLOF, lPOPE, and lFUS. A possible explanation for the occurrence of these excitations is that these ROIs were the ones that were anti-correlated to the stimulated nodes, when the network was in the resting-state.

### 3.4. Pathfinding in the brain

Besides simulation, another utility in BrainX^3^ is that it can be customized for real-time analysis and circuit extraction. This can be done either by analyzing output signals of neural activity from the simulation or by implementing graph-theoretic algorithms on the network. Here, we provide a example of bookmarking pathways efferent to the focal lesion discussed above. Bookmarking in BrainX^3^ can simply be done using natural gestures. In Figure 13 we trace the connectivity span (within the healthy dataset) of all the three areas that we had lesioned earlier. All edges emanating from the previously lesioned ROIs are bookmarked in thick black giving a clear spatial impression of the extent of the lesion on the network. Though the lesion lies only in the occipital and parietal lobes, its effects are felt as far as the frontal, temporal and limbic lobes. Extracting circuits this way is intuitive and user controllable, compared to automated processes based on correlation data.

## 4. Discussion

As techniques of quantitative analysis and measurement devices in neuroscience make improvements, it is becoming more evident that the role of large-scale dynamics and whole-brain measures cannot be ignored. Functional correlations by themselves are insufficient for inference of mechanisms and principles underlying brain function. Large-scale temporal activity maps across structurally connected brain areas are more informative of whole-brain circuit mechanisms. Being able to predict these maps by implementing realistic biophysical dynamics brings us a small step closer to identifying the neural correlates of cognitive functions. BrainX^3^ is a small step in this direction. It opens the possibility of analyzing neural activity propagation due to causal dynamics. Being immersive, it gives a much better intuitive anatomical perspective of the brain, than a 2D atlas would.

BrainX^3^, as we have shown in this paper, is a platform for data visualization, simulation, analysis and interaction, which combines computational power with human intuition in representing and interacting with large complex data. For the human connectome network above, we have shown an anatomically-spaced 3D simulation of whole-brain neural activity, based on the dynamical mean-field model, which was earlier tested in Deco et al. (2013) for resting-state dynamics. The results shown included the resting-state network, lesioned brains as well as externally stimulated networks. Our simulations above shed some insight on the spatial distribution of activity in the attractor state, how it maintains a level of resilience to damage, effects of noise and physiological perturbations. Specifically, we found that a noisy network seems to favor a low firing attractor. This is simply a consequence of the detailed biophysics of our model. Interestingly, both, computational and empirical studies in the literature have claimed that an increase in neural noise (in the form of random background activity) is associated with aging brains, which show a lower signal-to-noise ratio and less distinctive cortical representations leading to reduced information processing (Li et al., 2001; Li and Sikström, 2002; Hong and Rebec, 2012). In particular, fMRI data in D'Esposito et al. (1999), Huettel et al. (2001) show fewer activated voxels and an increase in noise in older participants, compared to younger ones. Our observation about the effect of noise on neural firing corroborates with the literature and as future research we plan to model neural dynamics in aging brains. We also found that a noisy network is less resilient to focal lesions. Between diffuse and focal lesions, the connectome network shows more resilience to the former, suggesting that the brain's wiring architecture is such that it provides alternate pathways for propagation of activity in order to protect against non-localized damage. Our results on TMS perturbations, generate temporal sequence of causal activations, which in the example of stimulating regions in the occipital and parietal lobes, map to efferent areas that presumably constitute a functional pathway. Interestingly, we also noticed that even though TMS inhibits most of the network, it also sparsely excites a few regions. Presumably, these are the regions anti-correlated to the perturbed ROIs. This suggests that even a lesioned network can show increased activity over sparsely distributed brain areas, compared to healthy brain networks. Knowledge of these active areas can be clinically useful for assessing levels of consciousness in patients with severe brain injury. These observations demonstrate the role of BrainX^3^ as a hypothesis generator. As is often the case with complex data, one might not always have a specific hypothesis to start with. Instead, discovering meaningful patterns and associations in big data might be a necessary incubation step for formulating well-defined hypotheses.

BrainX^3^ is not only a generator of simulated data of dynamical processes in complex networks, but it also provides a natural user interaction paradigm (including user immersion and gesture-based inputs) for the visualization and exploration of complex network datasets. In previous work, we have validated BrainX^3^ vs. standard desktop-based visualization and simulation tools and found that our system is better at structural understanding of the data based on the performance of subjects on a memory task (Betella et al., 2013, 2014a,b). As future applications of our technology, we foresee online user-interaction with simulations as a step toward virtual brain surgery, enabling a surgeon to try out several surgical procedures and assessing risk factors on models based on the patient's data before actually performing the surgery. However, to be useful for any form of precision surgery, besides improving usability and integration with other input/output devices relevant for surgery, the size of the simulation will have to be significantly scaled to much finer resolutions matching those of surgical standards and even more detailed biophysical models will have to be used (including plasticity and pharmacological inputs). This would mean working with networks having millions of nodes and proportionately many more connections (such as from precision microscopy), which would require optimizing BrainX^3^ with parallel computing. This is the next step in the development BrainX^3^, scaling and optimizing the simulation for very large networks.

## Author contributions

XA, RZ, AB, and PV contributed to the design, analysis, interpretation and writing of the manuscript. EM, PO, and DD contributed to the technical implementation. GD contributed to the analysis.

### Conflict of interest statement

The authors declare that the research was conducted in the absence of any commercial or financial relationships that could be construed as a potential conflict of interest.
